# Revealing mechanism of Caulis Sargentodoxae for the treatment of ulcerative colitis based on network pharmacology approach

**DOI:** 10.1042/BSR20204005

**Published:** 2021-01-29

**Authors:** Bin Liu, Xin Zheng, Jiajun Li, Xiong Li, Ruimei Wu, Jing Yang, Wei Liu, Gaoping Zhao

**Affiliations:** 1 Graduate School, Southwest Medical University, Luzhou, Sichuan 646000, China; 2 Department of General Surgery, The Second Affiliated Hospital of Chengdu Medical College, China National Nuclear Corporation 416 Hospital, Chengdu, Sichuan 610051, China; 3 The School of Biological Science and Technology, Chengdu Medical College, Chengdu 610083, China; 4 Department of Gastrointestinal Surgery, Sichuan Academy of Medical Sciences and Sichuan Provincial People’s Hospital, Chengdu, Sichuan 610072, China

**Keywords:** Caulis Sargentodoxae, Enrichment analysis, Network pharmacology, Ulcerative colitis

## Abstract

**Objective:** The traditional Chinese medicine Caulis Sargentodoxae is widely used in the treatment of ulcerative colitis (UC), but the mechanism remains unknown. The present study aims to reveal its effective components, targets and pathways through network pharmacology and bioinformatics approaches.

**Materials and methods:** Traditional Chinese Medicine Systems Pharmacology Database and Analysis Platform (TCMSP) was used to identify effective components. The ligand-based targets prediction was achieved through SwissTargetPrediction and TargetNet. UC-related targets were identified using Gene Expression Omnibus (GEO) data and DisGeNET. The common targets of disease and components were constructed and analyzed by PPI network. Lastly, Gene Ontology (GO) and Kyoto Encyclopedia of Genes and Genomes (KEGG) enrichment analyses are used to explain the functions of these common targets. Components-Targets-Pathways network was visualized and analyzed to further reveal the connection between the components and targets.

**Results:** Eight active components and 102 key targets were identified to play an important role in UC. These targets were related to regulation of protein serine/threonine kinase activity, positive regulation of cell motility, response to molecule of bacterial origin, response to toxic substance, ERK1 and ERK2 cascade, peptidyl-tyrosine modification, inositol lipid-mediated signaling, cellular response to drug, regulation of inflammatory response and leukocyte migration. Moreover, HIF-1 signaling pathway and PI3K-Akt signaling pathway were the key targets involved in UC-related signaling pathways.

**Conclusion:** The eight active components of Caulis Sargentodoxae mainly play a therapeutic role for UC through synergistic regulation of HIF-1 signaling pathway and PI3K-Akt signaling pathway.

## Introduction

Ulcerative colitis (UC) is a disease caused by a genetically predisposed host’s immune disorder to antigens in the intestine, and its incidence and prevalence are increasing globally [1,2]. The disease has the characteristics of remission and flare, and in most cases drugs are used to induce and maintain remission. However, up to 15% of cases require surgery due to medical treatment failure or inflammation that develops into dysplasia [3]. The primary treatment goal of UC is to achieve disease remission, prevent disease-related complications, surgery and tumor, and maintain the quality of life of patients [4]. The currently available data indicated that UC is an intestinal barrier disease, which is caused by epithelial cells or structural intestinal epithelial dysfunction, or strong inflammatory mediators and cells in the lamina propria to produce an inflammatory cascade. Therefore, treatment strategies can target epithelial cells or inflammatory cells to restore the intestinal barrier function and achieve clinical remission [5]. Currently, drugs used for UC include 5-aminosalicylic acid, steroids and immunosuppressants. However, long-term use of these drugs may cause a variety of side effects, such as liver and kidney toxicity, drug resistance and allergic reactions [6].

The use of herbs to treat various diseases has a long history. In addition, traditional Chinese medicine shows obvious advantages in the treatment of UC, such as low recurrence rate and fewer side effects [6]. Some natural active compounds have good therapeutic effects on experimental UC models. SM934 is a water-soluble artemisinin analog with anti-inflammatory and immunomodulatory effects. It was found to improve DSS-induced UC in mice by inhibiting neutrophils and macrophages [7]. Indigo Naturalis has been proven effective in a randomized, placebo-controlled trial [8]. However, there were potential adverse effects, including pulmonary hypertension [8]. This also reflects the complexity of Chinese herbal medicine.

**Figure 1:**
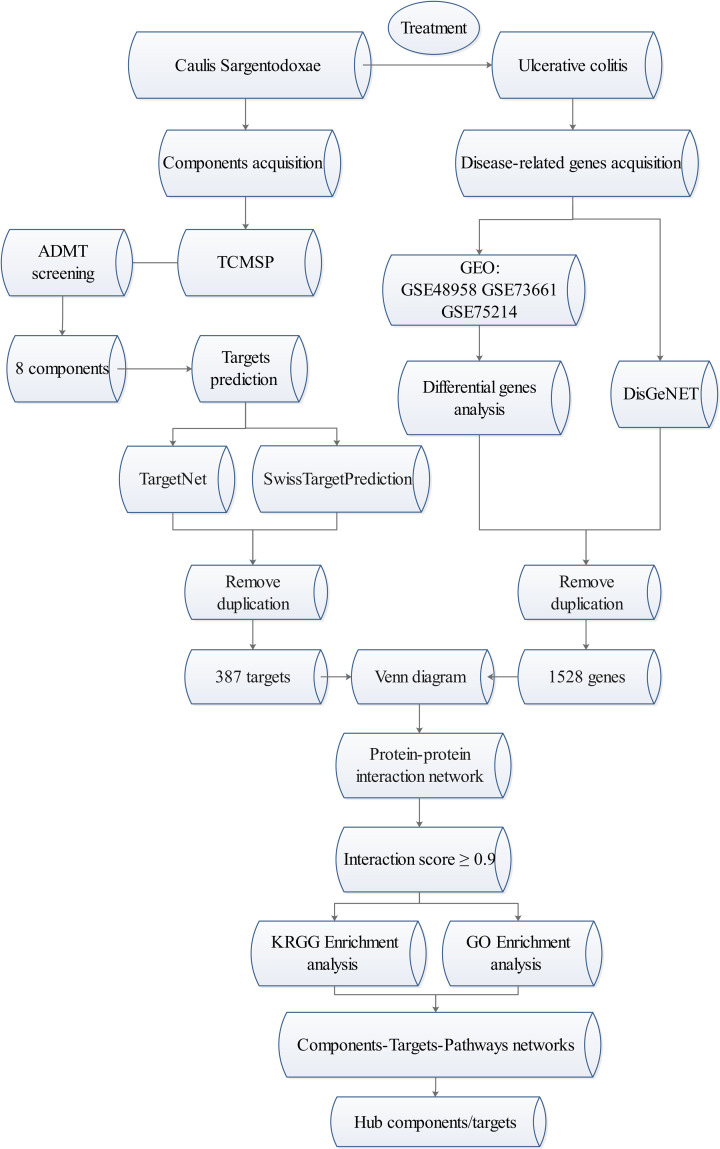
Work flow of the present study

Network pharmacology methods are rapidly evolving and applied to find new treatment opportunities and reposition of approved drugs. They can also effectively map the unexplored targets of natural products, thereby providing drug space for proteins related to various complex diseases [9]. The purpose of constructing the network is to realize the interaction between bioactive compounds and target proteins and the interaction between various target proteins, and then to identify and verify the key nodes through network analysis and verification [10]. Network pharmacology has been successfully used to reveal the active ingredients and mechanisms of indigo naturalis in the treatment of UC [11]. Caulis Sargentodoxae is also a high-frequency herb used in the treatment of UC. As a heat-clearing and detoxifying Chinese medicine, it has been found to have significant anti-inflammatory, anti-thrombotic, anti-tumor and anti-bacterial effects [12]. However, its active ingredients and targets are still unclear. The present study is based on the network pharmacology approaches and bioinformatics technologies to clarify the mechanism of Caulis Sargentodoxae in the treatment of UC, and to provide new candidate compounds for the treatment of UC. The entire workflow is shown in Figure 1.

## Materials and methods

### Components acquisition and screening

Traditional Chinese Medicine Systems Pharmacology Database and Analysis Platform (TCMSP) is a manually curated database containing 12144 herbal compounds and related pharmacokinetic properties (https://tcmspw.com/tcmsp.php) [13]. Oral bioavailability (OB), Drug-likeness (DL), Caco-2 permeability and other parameters information can be obtained from it. The components and characteristics of Caulis Sargentodoxae were retrieved from TCMSP. According to its suggested drug screening criteria, the components were screened. Finally, the compounds with molecular weight (MW) from 180 to 500 Dalton, OB ≥ 20%, DL ≥ 0.1 were retained.

### Targets collection of components in Caulis Sargentodoxae

Drug-targets prediction technology, which can predict component targets with high throughput and quickly, has been widely used in the preliminary research of drug development. The current prediction technologies mainly include ligands-based prediction methods (such as chemical similarity search, pharmacophore models etc.), targets-based prediction methods (such as reverse molecular docking) and machine learning methods. SwissTargetPrediction is a ligand-based targets prediction tool for bioactive small molecules (http://www.swisstargetprediction.ch/). The principle is to train the prediction model by multivariate logistic regression fitting of various size related subsets of known active molecules to weighted 2D and 3D similarity parameters [14]. TargetNet builds a large number of QSAR models based on current chemical and genomics data for molecular target prediction (http://targetnet.scbdd.com/calcnet/index/). Its server will predict the activity of user-submitted molecules in 623 human proteins by building a high-quality QSAR model for each human protein [15]. The structures and SMILES of each component were obtained from PubChem (https://pubchem.ncbi.nlm.nih.gov/). Subsequently, the SMILES of each component were uploaded to two databases for targets prediction.

### Acquisition of UC disease targets

Gene Expression Omnibus (GEO) is an ever-increasing platform for storing high-throughput sequencing data. Integrating sequencing information from different centers and performing gene differential expression analysis will facilitate the identification of disease treatment targets. We identified three series from GEO that all came from GPL6244, among which GSE73661 contains 23 active UC tissues and 12 normal tissues, GSE75214 contains 74 active UC tissues and 11 normal tissues, GSE48958 contains 7 active UC tissues and 8 normal tissues. Genes with adjusted *P*<0.05 and log2 (fold change) > 2 or log2 (fold change) < −2 were considered as differential genes, they have played an important role in the development of UC.

DisGeNET integrated data from public databases, GWASs, animal models and scientific literature, and collected a large number of variants and genes associated with human diseases (https://www.disgenet.org/). It currently covers more than 24000 diseases and traits, 17000 genes and 117000 genomic variants [16]. UC-related genes were also obtained via using ‘ulcerative colitis’ as the keyword to retrieve relevant genes in DisGeNET.

### Construction and analysis of target protein–protein interaction network

STRING (https://string-db.org/) can carry out direct (physical) and indirect (functional) interactions of proteins to achieve a comprehensive and objective network [17]. The targets were uploaded in the form of gene symbol, the species was selected as ‘*Homo sapiens*’, and the minimum required interaction score threshold ‘highest confidence (≥0.9)’ was set to obtain protein interaction information. Cytoscape v3.7.2 was used for visualization and in-depth analysis of the network. The hub nodes were identified in each network by calculating the size of the degree.

### Gene ontology and Kyoto Encyclopedia of Genes and Genomes signal pathway enrichment analysis

Gene ontology (GO) is a gene function classification system that comprehensively describes the attributes of genes and gene products of various species through dynamically updated GO terms. It is used to describe the biological process (BP), the cellular component (CC) and molecular function (MF) of gene sets of interest. Kyoto Encyclopedia of Genes and Genomes (KEGG) is a knowledge base for systematic analysis of gene function, which can describe pathway data involved in genes of interest, including connection of metabolism, membrane transport, signal transduction and cell cycle, etc. We used WebGestalt (http://www.webgestalt.org/#), a network enrichment analysis tool, for GO and KEGG enrichment analyses. The reference set was selected as human genome protein encoding. And the enrichment method was selected as over-representaion analysis.

### Construction and analysis of components-targets-pathways network

Components-Targets-Pathways network was constructed and visualized via Cytoscape v3.7.2 to identify key molecules and targets. The degrees of each component and target were calculated using CytoHubba.

### Toxicity prediction of components in Caulis Sargentodoxae

AdmetSAR 2.0 was a free tool and continually updated database for the prediction of ADMET properties, and has been widely used in chemical and pharmaceutical fields [18,19]. Each molecule’s SMILES was input to obtain organ toxicity and other toxic properties.

## Results

### Components of Caulis Sargentodoxae

Nine eligible valid results were screened through TCMSP database, with OB ≥ 20%, DL ≥ 0.1 and 500 ≥ MW ≥ 180 Dalton. These molecules have good OB and DL properties (Table 1 and Supplementary Figure S1). We have also made a toxicity prediction for compounds with SMILES. Analysis found that they have no carcinogenicity, but emodin and physcion may have hepatotoxicity. And physcion and (-)-catechin were positive in Ames mutagenesis (Supplementary Table S1).

**Table 1:** Parameter information of molecules in Caulis Sargentodoxae

TCMSP ID	Molecule name	Pubchem Cid	MW	OB (%)	DL
MOL000358	β-sitosterol	222284	414.79	36.91	0.75
MOL000359	Sitosterol	12303645	414.79	36.91	0.75
MOL000472	Emodin	3220	270.25	24.4	0.24
MOL000476	Physcion	10639	284.28	22.29	0.27
MOL007920	Meso-dihydroguaiaretic acid	476856	330.46	31.32	0.26
MOL007923	3-(4-Hydroxyphenyl)acrylic acid 4-hydroxyphenethyl ester	16119626	284.33	93.36	0.21
MOL007925	Glucosyringic acid	10383888	360.35	24.29	0.3
MOL000096	(-)-Catechin	73160	290.29	49.68	0.24
MOL007922	Syringaresnol diglucoside_qt	Not available	418.48	29.84	0.72

### Putative targets of components

**Figure 2:**
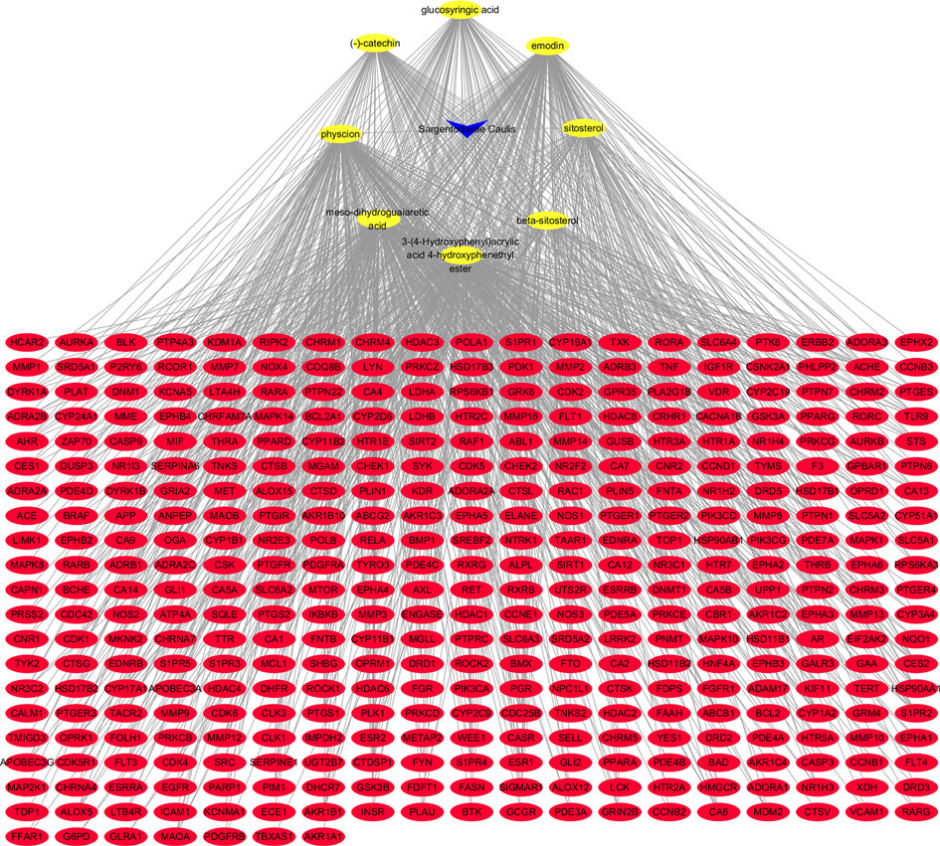
Components-targets-network of Caulis Sargentodoxae The yellow ellipses represent the components, and the red ellipses represent the targets of components in Caulis Sargentodoxae.

In both SwissTargetPrediction and TargetNet, we selected targets with a probability greater than 0 for subsequent analysis. Due to the lack of structural information and SMILES, we failed to obtain the targets of syringaresnol diglucoside_qt. The other 8 components get 396 targets through SwissTargetPrediction, and 719 targets through TargetNet. A total of 387 unique targets of 8 components were obtained after removal of the repeats. The relationship among the 8 components and the 387 targets was shown in Figure 2.

### UC-related targets

**Figure 3:**
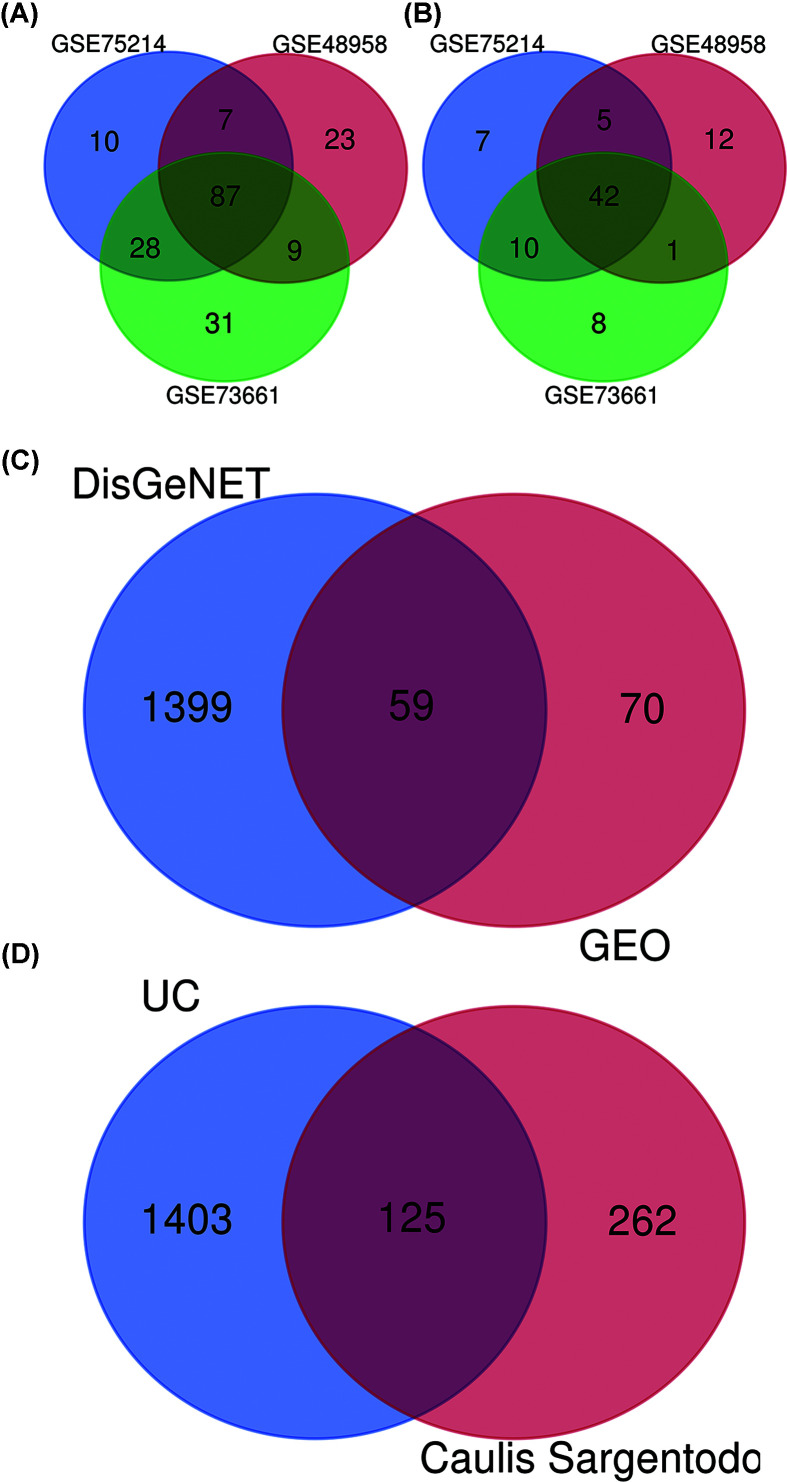
Work flow of targets screening (**A**) Venn diagram of up-regulated genes in UC from three GEO series. (**B**) Venn diagram of down-regulated genes in UC from three GEO series. (**C**) Venn diagram of targets of UC from differential genes (GEO) and DisGeNET. (**D**) Venn diagram of targets from UC and Caulis Sargentodoxae.

GSE73661, GSE75214 and GSE48958 were respectively analyzed for differential genes. Through the Venn diagram, we obtained 87 common up-regulated genes and 42 common down-regulated genes from three series (Figure 3A,B). In addition, we obtained 1458 UC-related targets from DisGeNET. A total of 1528 unique targets of UC were obtained after removal of the repeats (Figure 3C). By comparing the components targets and the disease targets through the Venn diagram, 125 targets for the treatment of UC were identified (Figure 3D). These targets may be the key proteins of Caulis Sargentodoxae in the treatment of UC.

### PPI network of 125 common targets

**Figure 4:**
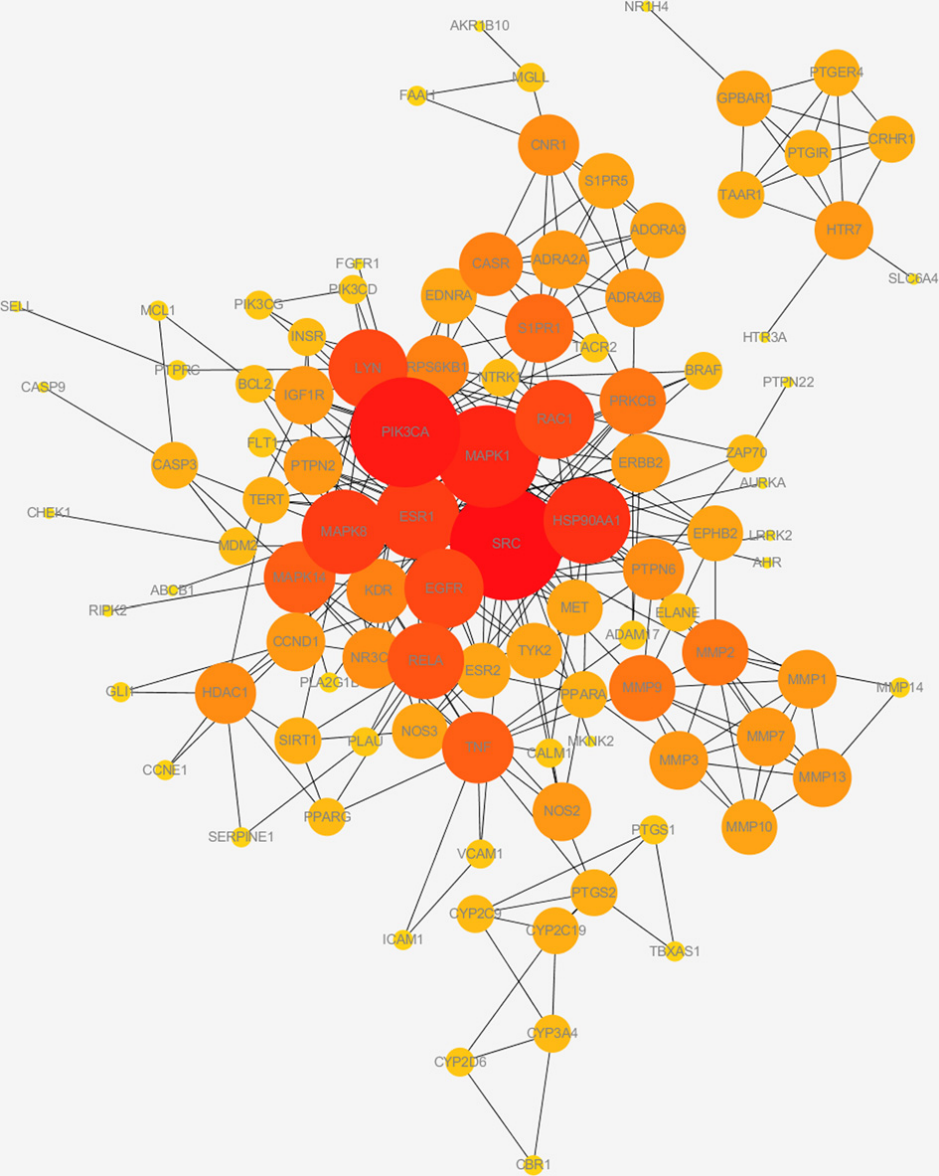
PPI network of common targets in UC and Caulis Sargentodoxae (interaction score ≥ 0.9) The darker nodes represent higher degrees.

We constructed PPI networks with threshold ‘highest confidence (≥0.9)’ in STRING. A network with 102 nodes and 618 edges was obtained (Figure 4). Based on the degree, we identified the key targets among them, including SRC, PIK3CA, MAPK1, HSP90AA1, MAPK8, ESR1, EGFR, LYN, RAC1, RELA, TNF, MAPK14, S1PR1, MMP2, MMP9, PRKCB (Table 2).

**Table 2:** Hub genes in PPI network

Rank	Name	Score
1	*SRC*	56
2	*PIK3CA*	54
3	*MAPK1*	48
4	*HSP90AA1*	36
5	*MAPK8*	34
5	*ESR1*	34
7	*EGFR*	30
7	*LYN*	30
7	*RAC1*	30
10	*RELA*	28
11	*TNF*	24
11	*MAPK14*	24
13	*S1PR1*	22
14	*MMP2*	20
14	*MMP9*	20
14	*PRKCB*	20

### GO and KEGG enrichment analyses

**Figure 5:**
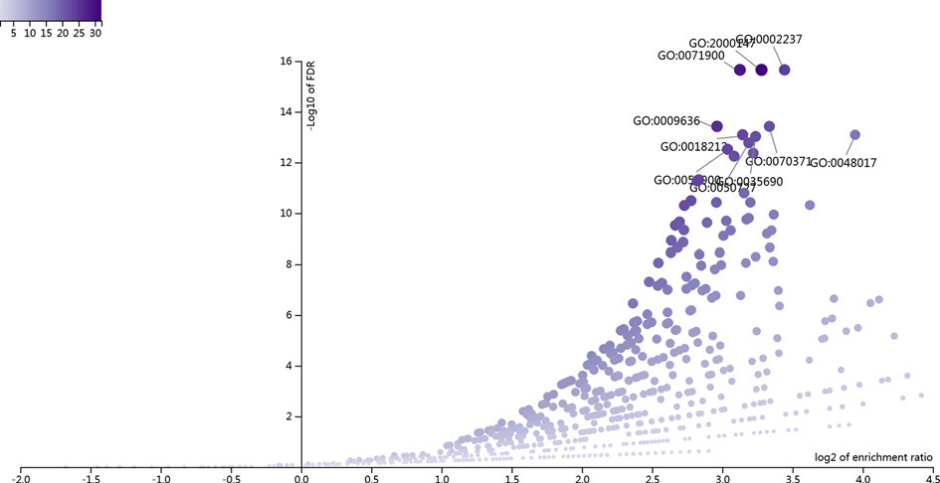
Volcano plot of the top ten significantly enriched GO terms of the common targets in UC and Caulis Sargentodoxae

**Figure 6:**
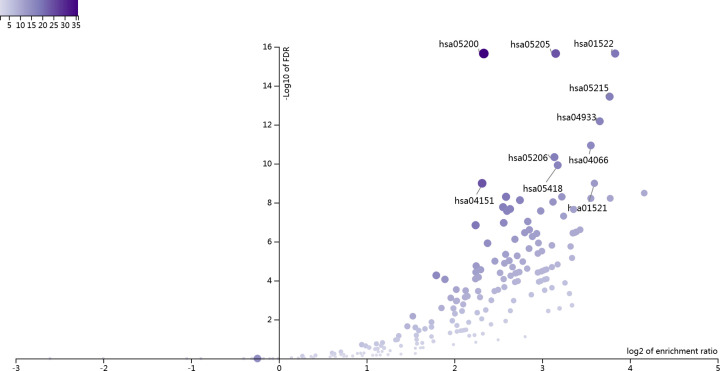
Volcano plot of the top ten significantly enriched pathways of the common targets in UC and Caulis Sargentodoxae

Using WebGestalt to conduct bioinformatics analysis of the above key targets. We identified the top ten GO terms and pathways that were significantly enriched, respectively (Figures 5 and 6). In UC, the BPs mainly regulated by Caulis Sargentodoxae were regulation of protein serine/threonine kinase activity, positive regulation of cell motility, response to molecule of bacterial origin, response to toxic substance, ERK1 and ERK2 cascade, peptidyl-tyrosine modification, inositol lipid-mediated signaling, cellular response to drug, regulation of inflammatory response and leukocyte migration (Table 3).

**Table 3:** Top ten significantly enriched GO terms of key targets

Gene set	Description	Size	Expect	Ratio	*P*-value	FDR
GO:0071900	Regulation of protein serine/threonine kinase activity	497	3.323	8.7272	0	0
GO:2000147	Positive regulation of cell motility	493	3.2962	9.7081	0	0
GO:0002237	Response to molecule of bacterial origin	330	2.2064	10.878	0	0
GO:0009636	Response to toxic substance	499	3.3363	7.793	2.22E-16	3.77E-14
GO:0070371	ERK1 and ERK2 cascade	326	2.1796	10.093	2.22E-16	3.77E-14
GO:0018212	Peptidyl-tyrosine modification	389	2.6009	8.8432	6.66E-16	8.09E-14
GO:0048017	Inositol lipid-mediated signaling	165	1.1032	15.41	6.66E-16	8.09E-14
GO:0035690	Cellular response to drug	349	2.3334	9.4282	8.88E-16	9.44E-14
GO:0050727	Regulation of inflammatory response	361	2.4137	9.1148	1.78E-15	1.68E-13
GO:0050900	Leukocyte migration	419	2.8014	8.21	3.55E-15	3.02E-13

In UC, the pathways mainly regulated by Caulis Sargentodoxae were Pathways in cancer, Proteoglycans in cancer, Endocrine resistance, Prostate cancer, AGE-RAGE signaling pathway in diabetic complications, HIF-1 signaling pathway, MicroRNAs in cancer, Fluid shear stress and atherosclerosis, PI3K-Akt signaling pathway and EGFR tyrosine kinase inhibitor resistance (Table 4). Among them, the changes of HIF-1 signaling pathway and PI3K-Akt signaling pathway are most closely related to UC.

**Table 4:** Top ten significantly enriched pathways of key targets

Gene set	Description	Size	Expect	Ratio	*P*-value	FDR
hsa05200	Pathways in cancer	524	7.1346	5.0458	0	0
hsa05205	Proteoglycans in cancer	198	2.6959	8.9024	0	0
hsa01522	Endocrine resistance	98	1.3343	14.239	0	0
hsa05215	Prostate cancer	97	1.3207	13.629	4.44E-16	3.62E-14
hsa04933	AGE-RAGE signaling pathway in diabetic complications	99	1.348	12.612	1.02E-14	6.66E-13
hsa04066	HIF-1 signaling pathway	100	1.3616	11.751	2.13E-13	1.16E-11
hsa05206	MicroRNAs in cancer	150	2.0424	8.8133	9.90E-13	4.61E-11
hsa05418	Fluid shear stress and atherosclerosis	138	1.879	9.0475	2.93E-12	1.20E-10
hsa04151	PI3K-Akt signaling pathway	354	4.82	4.9793	3.10E-11	1.02E-09
hsa01521	EGFR tyrosine kinase inhibitor resistance	79	1.0756	12.086	3.11E-11	1.02E-09

### Components-targets-pathways network

The key targets of Caulis Sargentodoxae enriched to HIF-1 signaling pathway were BCL2, EGFR, ERBB2, FLT1, IGF1R, INSR, MAPK1, MKNK2, NOS2, NOS3, PIK3CA, PIK3CD, PRKCB, RELA, RPS6KB1 and SERPINE1 (Supplementary Figure S2). The key targets of Caulis Sargentodoxae enriched to PI3K-Akt signaling pathway were BCL2, CASP, CCND1, CCNE1, EGFR, ERBB2, FGFR1, FLT1, HSP90AA1, IGF1R, INSR, KDR, MAPK1, MCL1, MDM2, MET, NOS3, NTRK1, PIK3CA, PIK3CD, PIK3CG, RAC1, RELA, RPS6KB1 (Supplementary Figure S3). In the PPI network, EGFR, PRKCB, MAPK1, HSP90AA1, RELA, RAC1, PIK3CA were also identified as hub genes.

**Figure 7:**
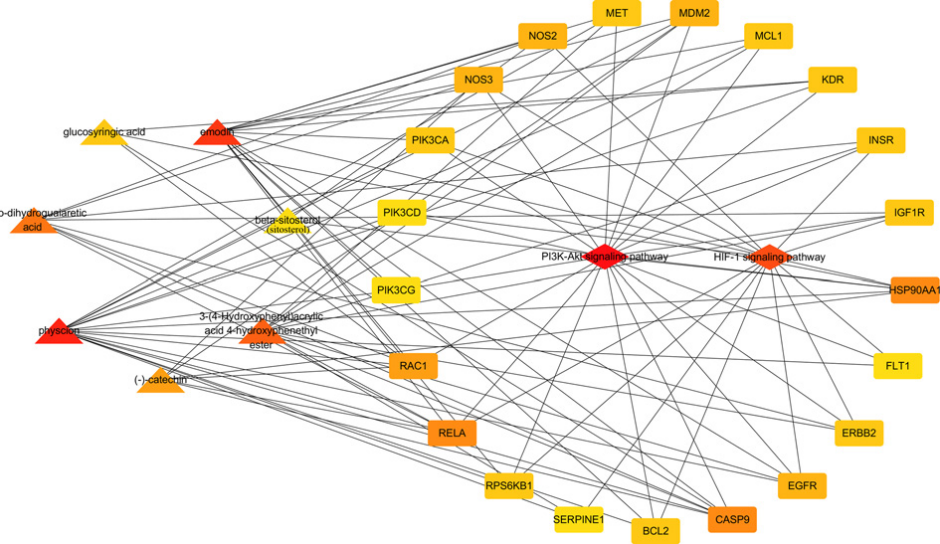
Components-targets-pathways network of Caulis Sargentodoxae The triangles represent the components, the quadrangles represent the pathways, and the squarenesses represent the targets that can be regulated by the components and are in the pathways. The darker nodes represent higher degrees.

Moreover, we constructed components-targets-pathways network with 8 components, 102 hub targets and HIF-1 signaling pathway and PI3K-Akt signaling pathway (Figure 7). The network showed that these eight molecules were involved in regulating HIF-1 signaling pathway and PI3K-Akt signaling pathway. In this network, we further identified key molecules and targets using degrees (Table 5). Physcion, emodin, 3-(4-Hydroxyphenyl)acrylic acid 4-hydroxyphenethyl ester and meso-dihydroguaiaretic acid were the most important components that regulates the above two pathways.

**Table 5:** Hub genes/molecules in components-targets-pathways network

Rank	Name	Score
1	Physcion	18
2	Emodin	17
3	3-(4-Hydroxyphenyl)acrylic acid 4-hydroxyphenethyl ester	12
4	Meso-dihydroguaiaretic acid	9
5	CASP9	7
5	RELA	7
5	HSP90AA1	7
8	(-)-Catechin	6
8	RAC1	6
10	MDM2	5
10	NOS3	5
10	NOS2	5
10	EGFR	5
14	MET	4
14	BCL2	4
14	PIK3CA	4
14	Glucosyringic acid	4
14	RPS6KB1	4
14	ERBB2	4
14	KDR	4
14	MCL1	4
14	INSR	4
14	IGF1R	4
24	β-sitosterol	3
24	PIK3CG	3
24	PIK3CD	3
24	SERPINE1	3
24	FLT1	3

## Discussion

Although drugs such as aminosalicylate, glucocorticoids, antibiotics and anti-TNFα play a good role in the treatment of UC, they still fail to achieve healing results and often cause side effects [20]. In China, traditional Chinese medicine prescriptions are widely used to treat recurrent UC. The mechanism of these drug combination based on the basic theory of traditional Chinese medicine is often difficult to elucidate due to the complexity of the components. The holistic view of Chinese medicine has a lot in common with the key ideas of network pharmacology and systems biology. This network-based approach can explain the combination rules and network regulation effects of herbal formulas [21]. Network pharmacology strategies were successfully used to reveal mechanism of Lang Chuang Wan in treating systemic lupus erythematosus [22] and Yizhiqingxin formula on Alzheimer’s disease [23].

Combined with the bioinformatics research on diseases, we can more clearly define the targets and specific mechanisms of Chinese medicine on the diseases, and identify key active molecules. This is of great significance to the development of natural medicines and the treatment of diseases. In our study, we successfully identified eight active molecules and two important pathways related to UC through network pharmacology and bioinformatics. They played a synergistic role in regulating HIF-1 signaling pathway and PI3K-Akt signaling pathway. Current evidences showed that Treg/Th17 imbalance is closely related to UC. The mTOR-HIF-1α-Th17 and STAT3-HIF-1α-Th17 pathways play an important role in the development of Th17 and the activation of IL-17. In addition, HIF-1α can bind to Foxp3, thereby affecting the development and function of Treg [24]. PI3K can respond to LPS and TNF-α, thereby activating Akt and affecting NF-κB to produce anti-apoptotic and pro-inflammatory effects [25]. Therefore, Caulis Sargentodoxae can improve UC by regulating them.

According to Components-Targets-Pathways network, the degrees of physcion, emodin, 3-(4-Hydroxyphenyl)acrylic acid 4-hydroxyphenethyl ester and meso-dihydroguaiaretic acid were the highest. Physcion is an anthraquinone compound commonly found in various plants, and shows anti-cancer properties in a variety of tumors. It can significantly inhibit the production of LPS/IFN-γ-induced NO in various cells via attenuating iNOS [26]. It can also reduce the gene expression of TNF-α, IL-6 and IL-1β in hepatocellular carcinoma cells induced by LPS in a dose-dependent manner by regulating p38 [27]. This indicated that it has good anti-inflammatory ability. And in our study, it was also identified as the most critical component for the treatment of UC. Emodin is a natural anthraquinone derivative with a wide range of pharmacological properties, including anticancer, liver protection, anti-inflammatory, antioxidant and antibacterial activities [28]. Emodin was found to induce autophagy in L02 human hepatocytes by inhibiting PI3K/AKT/mTOR signaling pathway [29]. Emodin was found to protect mice from DSS-induced colitis via the regulation of TLR5/NF-κB signaling pathway [30], and significantly inhibit LPS-mediated NF-κB activation and DNA binding activity in RAW264.7 cells [31]. This is consistent with our computer-based research, and indicates that emodin can regulate PI3K-Akt signaling pathway to exert anti-inflammatory effects. Meso-dihydroguaiaretic acid was found can attenuate airway inflammation and mucus hypersecretion in an ovalbumin-induced murine model of asthma via inhibiting the activation of NF-κB, ERK1/2 and p38 [32]. However, the pharmacological activity of 3-(4-Hydroxyphenyl)acrylic acid 4-hydroxyphenethyl ester has not been reported yet, and further experiments are needed.

Overall, our results suggested that Caulis Sargentodoxae can treat UC through regulating HIF-1 signaling pathway and PI3K-Akt signaling pathway, but this effect is a synergistic effect of eight active components. It is necessary to further compare the therapeutic effects of Caulis Sargentodoxae and its different components in animal models, and further evaluate these molecules in terms of safety and higher efficacy. Analyzing and verifying the effective dose and toxicity of different molecules by using pharmacology and toxicology experiments will also have important significance for clinical applications.

## Supplementary Material

Supplementary Figures S1-S3 and Supplementary Table S1

## Data Availability

The data used to support the findings of the present study are included within the article and the supplementary files.

## Abbreviations

BP: biological process
DL: drug-likeness
DSS: dextran sulphate sodium
EGFR: epidermal growth factor receptor
GEO: gene expression omnibus
GO: gene ontology
GWAS: genome-wide association studies
IFN: interferon
IL: interleukin
iNOS: inducible nitric oxide synthase
KEGG: Kyoto Encyclopedia of Genes and Genomes
LPS: lipopolysaccharide
MW: molecular weight
OB: oral bioavailability
PPI: protein-protein interaction
TCMSP: Traditional Chinese Medicine Systems Pharmacology Database and Analysis Platform
Th17: T helper cell 17
TLR5: toll like receptor 5
Treg: regulatory T cell
UC: ulcerative colitis

## References

[1] Ungaro R., Mehandru S., Allen P.B., Peyrin-Biroulet L. and Colombel J.F. (2017) Ulcerative colitis. Lancet 389, 1756–1770 10.1016/S0140-6736(16)32126-2 27914657 PMC6487890

[2] Hindryckx P., Jairath V. and D’Haens G. (2016) Acute severe ulcerative colitis: from pathophysiology to clinical management. Nat. Rev. Gastroenterol. Hepatol. 13, 654–664 10.1038/nrgastro.2016.116 27580687

[3] Feuerstein J.D., Moss A.C. and Farraye F.A. (2019) Ulcerative colitis. Mayo Clin. Proc. 94, 1357–1373 10.1016/j.mayocp.2019.01.018 31272578

[4] Kayal M. and Shah S. (2019) Ulcerative colitis: current and emerging treatment strategies. J. Clin. Med. 9, 10.3390/jcm9010094 31905945 PMC7019865

[5] Kobayashi T., Siegmund B., Le Berre C., Wei S.C., Ferrante M., Shen B. et al. (2020) Ulcerative colitis. Nat. Rev. Dis. Primers 6, 74 10.1038/s41572-020-0205-x 32913180

[6] Cao S.Y., Ye S.J., Wang W.W., Wang B., Zhang T. and Pu Y.Q. (2019) Progress in active compounds effective on ulcerative colitis from Chinese medicines. Chin. J. Nat. Med. 17, 81–102 10.1016/S1875-5364(19)30012-3 30797423

[7] Yan Y.X., Shao M.J., Qi Q., Xu Y.S., Yang X.Q., Zhu F.H. et al. (2018) Artemisinin analogue SM934 ameliorates DSS-induced mouse ulcerative colitis via suppressing neutrophils and macrophages. Acta Pharmacol. Sin. 39, 1633–1644 10.1038/aps.2017.185 29849131 PMC6289314

[8] Naganuma M., Sugimoto S., Mitsuyama K., Kobayashi T., Yoshimura N., Ohi H. et al. (2018) Efficacy of indigo naturalis in a multicenter randomized controlled trial of patients with ulcerative colitis. Gastroenterology 154, 935–947 10.1053/j.gastro.2017.11.024 29174928

[9] Kibble M., Saarinen N., Tang J., Wennerberg K., Mäkelä S. and Aittokallio T. (2015) Network pharmacology applications to map the unexplored target space and therapeutic potential of natural products. Nat. Prod. Rep. 32, 1249–1266 10.1039/C5NP00005J 26030402

[10] Luo T.T., Lu Y., Yan S.K., Xiao X., Rong X.L. and Guo J. (2020) Network pharmacology in research of chinese medicine formula: methodology, application and prospective. Chinese J. Integr. Med. 26, 72–80 10.1007/s11655-019-3064-0 30941682

[11] Gu S., Xue Y., Gao Y., Shen S., Zhang Y., Chen K. et al. (2020) Mechanisms of indigo naturalis on treating ulcerative colitis explored by GEO gene chips combined with network pharmacology and molecular docking. Sci. Rep. 10, 15204 10.1038/s41598-020-71030-w 32938944 PMC7495487

[12] Yang L., Yin P., Cao X. and Liu Y. (2019) Screen for potential candidate alternatives of sargentodoxa cuneata from its six adulterants based on their phenolic compositions and antioxidant activities. Int. J. Mol. Sci. 20, 10.3390/ijms20215427 PMC6862427 31683574

[13] Ru J., Li P., Wang J., Zhou W., Li B., Huang C. et al. (2014) TCMSP: a database of systems pharmacology for drug discovery from herbal medicines. J. Cheminformatics 6, 13 10.1186/1758-2946-6-13 24735618 PMC4001360

[14] Daina A., Michielin O. and Zoete V. (2019) SwissTargetPrediction: updated data and new features for efficient prediction of protein targets of small molecules. Nucleic Acids Res. 47, W357–W364 10.1093/nar/gkz382 31106366 PMC6602486

[15] Yao Z.J., Dong J., Che Y.J., Zhu M.F., Wen M., Wang N.N. et al. (2016) TargetNet: a web service for predicting potential drug-target interaction profiling via multi-target SAR models. J. Comput. Aided Mol. Des. 30, 413–424 10.1007/s10822-016-9915-2 27167132

[16] Piñero J., Ramírez-Anguita J.M., Saüch-Pitarch J., Ronzano F., Centeno E., Sanz F. et al. (2020) The DisGeNET knowledge platform for disease genomics: 2019 update. Nucleic Acids Res. 48, D845–D855 31680165 10.1093/nar/gkz1021 PMC7145631

[17] Szklarczyk D., Gable A.L., Lyon D., Junge A., Wyder S., Huerta-Cepas J. et al. (2019) STRING v11: protein-protein association networks with increased coverage, supporting functional discovery in genome-wide experimental datasets. Nucleic Acids Res. 47, D607–D613 10.1093/nar/gky1131 30476243 PMC6323986

[18] Cheng F., Li W., Zhou Y., Shen J., Wu Z., Liu G. et al. (2012) admetSAR: a comprehensive source and free tool for assessment of chemical ADMET properties. J. Chem. Inf. Model. 52, 3099–3105 10.1021/ci300367a 23092397

[19] Yang H., Lou C., Sun L., Li J., Cai Y., Wang Z. et al. (2019) admetSAR 2.0: web-service for prediction and optimization of chemical ADMET properties. Bioinformatics 35, 1067–1069 10.1093/bioinformatics/bty707 30165565

[20] Cao F., Liu J., Sha B.X. and Pan H.F. (2019) Natural products: experimental efficient agents for inflammatory bowel disease therapy. Curr. Pharm. Des. 25, 4893–4913 10.2174/1381612825666191216154224 31840596

[21] Li S. and Zhang B. (2013) Traditional Chinese medicine network pharmacology: theory, methodology and application. Chinese J. Nat. Med. 11, 110–120 10.1016/S1875-5364(13)60037-0 23787177

[22] Gao Y., Wang K.X., Wang P., Li X., Chen J.J., Zhou B.Y. et al. (2020) A novel network pharmacology strategy to decode mechanism of Lang Chuang Wan in treating systemic lupus erythematosus. Front. Pharmacol. 11, 512877 10.3389/fphar.2020.512877 33117150 PMC7562735

[23] Zhang T., Pan L., Cao Y., Liu N., Wei W. and Li H. (2020) Identifying the mechanisms and molecular targets of Yizhiqingxin formula on Alzheimer’s disease: coupling network pharmacology with GEO database. Pharmacogenom. Personal. Med. 13, 487–502 10.2147/PGPM.S269726 33116763 PMC7571582

[24] Yao J., Wei C., Wang J.Y., Zhang R., Li Y.X. and Wang L.S. (2015) Effect of resveratrol on Treg/Th17 signaling and ulcerative colitis treatment in mice. World J. Gastroenterol. 21, 6572–6581 10.3748/wjg.v21.i21.6572 26074695 PMC4458767

[25] Wei J. and Feng J. (2010) Signaling pathways associated with inflammatory bowel disease. Recent Patents on Inflammation & Allergy Drug Discovery 4, 105–117 10.2174/187221310791163071 20001899

[26] Li X., Liu Y., Chu S., Yang S., Peng Y., Ren S. et al. (2019) Physcion and physcion 8-O-β-glucopyranoside: A review of their pharmacology, toxicities and pharmacokinetics. Chem. Biol. Interact. 310, 108722 10.1016/j.cbi.2019.06.035 31226286

[27] Selim N.M., Elgazar A.A., Abdel-Hamid N.M., El-Magd M.R.A., Yasri A., Hefnawy H.M.E. et al. (2019) Chrysophanol, Physcion, Hesperidin and Curcumin modulate the gene expression of pro-inflammatory mediators induced by LPS in HepG2: in silico and molecular studies. Antioxidants 8, 10.3390/antiox8090371 PMC6770650 31484451

[28] Dong X., Fu J., Yin X., Cao S., Li X., Lin L. et al. (2016) Emodin: a review of its pharmacology, toxicity and pharmacokinetics. Phytother. Res. 30, 1207–1218 10.1002/ptr.5631 27188216 PMC7168079

[29] Zheng X.Y., Yang S.M., Zhang R., Wang S.M., Li G.B. and Zhou S.W. (2019) Emodin-induced autophagy against cell apoptosis through the PI3K/AKT/mTOR pathway in human hepatocytes. Drug Des. Dev. Ther. 13, 3171–3180 10.2147/DDDT.S204958 31564833 PMC6734549

[30] Luo S., Deng X., Liu Q., Pan Z., Zhao Z., Zhou L. et al. (2018) Emodin ameliorates ulcerative colitis by the flagellin-TLR5 dependent pathway in mice. Int. Immunopharmacol. 59, 269–275 10.1016/j.intimp.2018.04.010 29669309

[31] Zhu T., Zhang W., Feng S.J. and Yu H.P. (2016) Emodin suppresses LPS-induced inflammation in RAW264.7 cells through a PPARγ-dependent pathway. Int. Immunopharmacol. 34, 16–24 10.1016/j.intimp.2016.02.014 26910236

[32] Song J.W., Seo C.S., Cho E.S., Kim T.I., Won Y.S., Kwon H.J. et al. (2016) meso-Dihydroguaiaretic acid attenuates airway inflammation and mucus hypersecretion in an ovalbumin-induced murine model of asthma. Int. Immunopharmacol. 31, 239–247 10.1016/j.intimp.2015.12.033 26773771

