# Supplementary material for: Revealing mechanism of Caulis Sargentodoxae for the treatment of ulcerative colitis based on network pharmacology approach
Source: Biosci Rep. 2021 Jan 29;41(1):BSR20204005. doi: 10.1042/BSR20204005 (PMC7846960; doi:10.1042/BSR20204005)
Supplement: Supplementary Figures S1-S3 and Supplementary Table S1 [file BSR-2020-4005_supp.pdf]

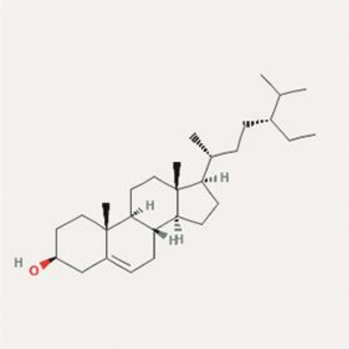

beta-sitosterol

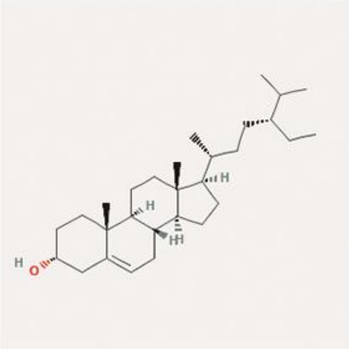

sitosterol

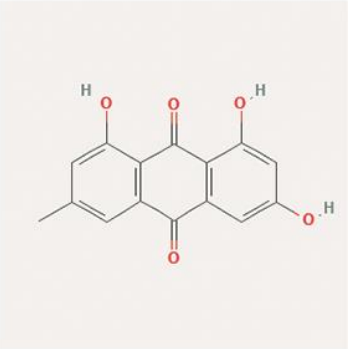

emodin

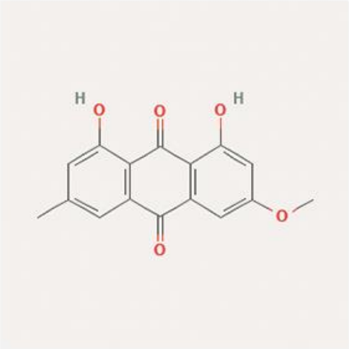

physcion

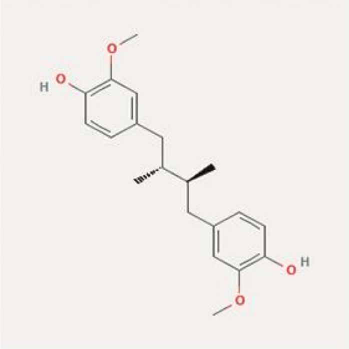

meso-dihydroguaiaretic acid

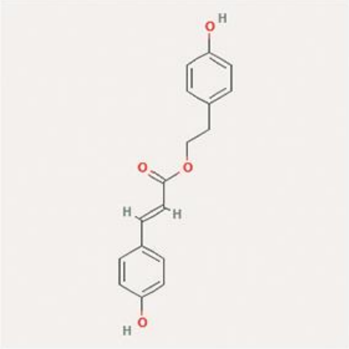

3-(4-Hydroxyphenyl)acrylic acid 4-hydroxyphenethyl ester

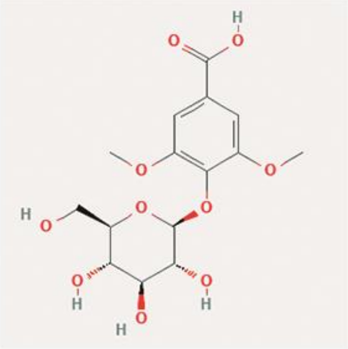

glucosyringic acid

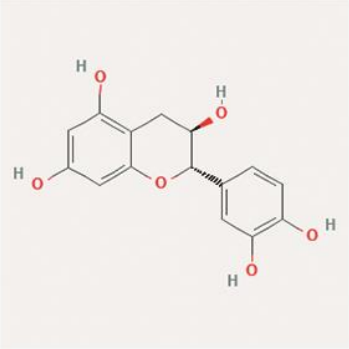

(-)-catechin

Supplementary Figure 1. Chemical structures of the components in Caulis Sargentodoxae.

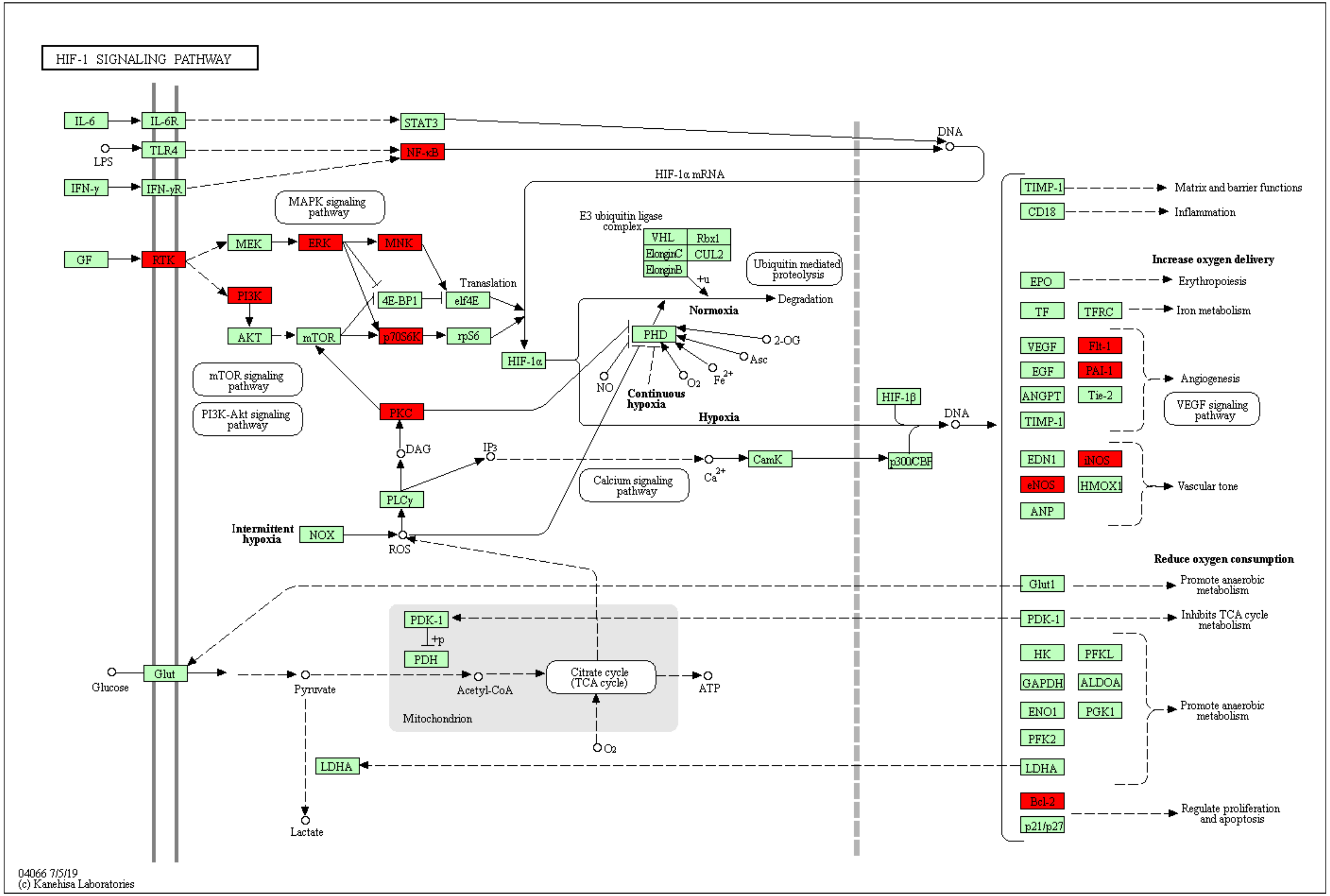
HIF-1 SIGNALING PATHWAY
IL-6
IL-6R
STAT3
LPS
TLR4
NF-κB
IFN-γ
IFN-γR
DNA
HIF-1α mRNA
MAPK signaling pathway
GF
RTK
MEK
ERK
MNK
PI3K
AKT
mTOR
4E-BP1
eIF4E
Translation
p70S6K
rpS6
HIF-1α
mTOR signaling pathway
PI3K-Akt signaling pathway
PKC
DAG
IP3
PLCγ
Intermittent hypoxia
NOX
ROS
E3 ubiquitin ligase complex
VHL
Rbx1
ElonginC
CUL2
ElonginB
+u
Ubiquitin mediated proteolysis
Degradation
Normoxia
PHD
2-OG
Asc
O2
Fe2+
NO
Continuous hypoxia
Hypoxia
Calcium signaling pathway
Ca2+
CamK
HIF-1β
p300/CBP
TIMP-1
Matrix and barrier functions
CD18
Inflammation
Increase oxygen delivery
EPO
Erythropoiesis
TF
TFRC
Iron metabolism
VEGF
Flt-1
EGF
PAI-1
ANGPT
Tie-2
Angiogenesis
VEGF signaling pathway
EDN1
iNOS
eNOS
HMOX1
Vascular tone
ANP
Reduce oxygen consumption
Glut1
Promote anaerobic metabolism
PDK-1
Inhibits TCA cycle metabolism
PDH
+p
Glucose
Glut
Pyruvate
Acetyl-CoA
Citrate cycle (TCA cycle)
ATP
Mitochondrion
O2
LDHA
Lactate
HK
PFKL
GAPDH
ALDOA
ENO1
PGK1
PFK2
Bcl-2
p21/p27
Regulate proliferation and apoptosis
04066 7/5/19
(c) Kanehisa Laboratories

Supplementary Figure 2. HIF-1 signaling pathway(KEGG). Red nodes represent the targets regulated by Caulis Sargentodoxae in UC.

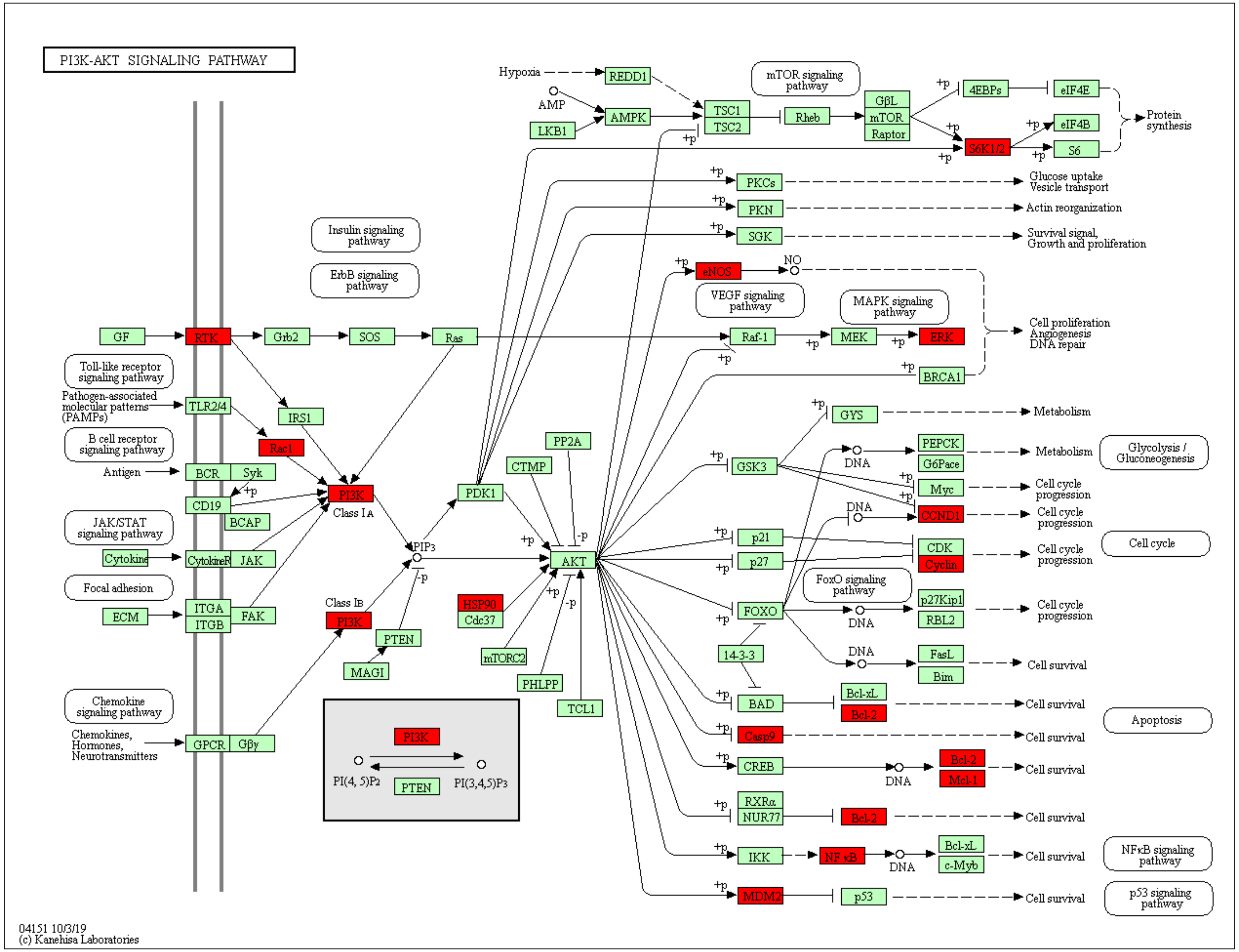
PI3K-AKT SIGNALING PATHWAY
Hypoxia
REDD1
AMP
AMPK
LKB1
TSC1
TSC2
mTOR signaling pathway
Rheb
GβL
mTOR
Raptor
4EBPs
eIF4E
eIF4B
S6K1/2
S6
Protein synthesis
PKCs
Glucose uptake
Vesicle transport
PKN
Actin reorganization
SGK
Survival signal, Growth and proliferation
Insulin signaling pathway
ErbB signaling pathway
eNOS
NO
VEGF signaling pathway
MAPK signaling pathway
Cell proliferation
Angiogenesis
DNA repair
GF
RTK
Grb2
SOS
Ras
Raf-1
MEK
ERK
BRCA1
Toll-like receptor signaling pathway
Pathogen-associated molecular patterns (PAMPs)
TLR2/4
IRS1
Rac1
B cell receptor signaling pathway
Antigen
BCR
Syk
CD19
BCAP
PI3K
Class IA
JAK/STAT signaling pathway
Cytokine
CytokineR
JAK
Focal adhesion
ECM
ITGA
ITGB
FAK
Chemokine signaling pathway
Chemokines, Hormones, Neurotransmitters
GPCR
Gβγ
Class IB
PI3K
PTEN
MAGI
PIP3
PDK1
CTMP
PP2A
AKT
HSP90
Cdc37
mTORC2
PHLPP
TCL1
PI3K
PI(4,5)P2
PTEN
PI(3,4,5)P3
GYS
Metabolism
GSK3
DNA
PEPCK
G6Pace
Metabolism
Glycolysis / Gluconeogenesis
Myc
Cell cycle progression
CCND1
Cell cycle progression
p21
p27
CDK
Cyclin
Cell cycle progression
Cell cycle
FoxO signaling pathway
FOXO
p27Kip1
RBL2
Cell cycle progression
14-3-3
FasL
Bim
Cell survival
BAD
Bcl-xL
Bcl-2
Cell survival
Apoptosis
Casp9
Cell survival
CREB
Bcl-2
Mcl-1
Cell survival
RXRα
NUR77
Bcl-2
Cell survival
IKK
NF-κB
Bcl-xL
c-Myb
Cell survival
NFκB signaling pathway
MDM2
p53
Cell survival
p53 signaling pathway
04151 10/3/19
(c) Kanehisa Laboratories

Supplementary Figure 3. PI3K-Akt signaling pathway(KEGG). Red nodes represent the targets regulated by Caulis Sargentodoxae in UC.

Supplementary Table 1. Toxicity prediction of 8 active components in Caulis Sargentodoxae

Molecule Name	Carcinogenicity (binary)	Carcinogenicity (trinary)	Ames mutagenesis	Hepatotoxicity	Acute Oral Toxicity
beta-sitosterol	-	Non-required	-	-	3.059kg/mol
sitosterol	-	Non-required	-	-	3.059kg/mol
emodin	-	Non-required	-	+	2.564kg/mol
physcion	-	Non-required	+	+	2.665kg/mol
meso-dihydroguaiaretic acid	-	Non-required	-	-	1.969kg/mol
3-(4-Hydroxyphenyl)acrylic acid 4-hydroxyphenethyl ester	-	Non-required	-	-	1.904kg/mol
glucosyringic acid	-	Non-required	-	-	2.369kg/mol
(-)-catechin	-	Non-required	+	-	1.972kg/mol
